# Comparative Transcriptomics and Metabolomics Analyses of *Avicennia marina* and *Kandelia obovata* under Chilling Stress during Seedling Stage

**DOI:** 10.3390/ijms242316989

**Published:** 2023-11-30

**Authors:** Shu-Min Wang, You-Shao Wang, Hao Cheng

**Affiliations:** 1 State Key Laboratory of Tropical Oceanography, South China Sea Institute of Oceanology, Chinese Academy of Sciences, Guangzhou 510301, China; wangshumin202@mails.ucas.ac.cn (S.-M.W.); chenghao@scsio.ac.cn (H.C.); 2 Daya Bay Marine Biology Research Station, Chinese Academy of Sciences, Shenzhen 518121, China; 3 Innovation Academy of South China Sea Ecology and Environmental Engineering, Chinese Academy of Sciences, Guangzhou 510301, China; 4 University of Chinese Academy of Sciences, Beijing 100049, China

**Keywords:** mangrove, chilling stress, transcriptome sequence, *MAPK* cascade, phenylpropanoid metabolism

## Abstract

One of the most productive ecosystems in the world, mangroves are susceptible to cold stress. However, there is currently insufficient knowledge of the adaptation mechanisms of mangrove plants in response to chilling stress. This study conducted a comparative analysis of transcriptomics and metabolomics to investigate the adaptive responses of *Kandelia obovata* (chilling-tolerant) and *Avicennia marina* (chilling-sensitive) to 5 °C. The transcriptomics results revealed that differentially expressed genes (DEGs) were mostly enriched in signal transduction, photosynthesis-related pathways, and phenylpropanoid biosynthesis. The expression pattern of genes involved in photosynthesis-related pathways in *A. marina* presented a downregulation of most DEGs, which correlated with the decrease in total chlorophyll content. In the susceptible *A. marina*, all DEGs encoding mitogen-activated protein kinase were upregulated. Phenylpropanoid-related genes were observed to be highly induced in *K. obovata*. Additionally, several metabolites, such as 4-aminobutyric acid, exhibited higher levels in *K. obovata* than in *A. marina*, suggesting that chilling-tolerant varieties regulated more metabolites in response to chilling. The investigation defined the inherent distinctions between *K. obovata* and *A. marina* in terms of signal transduction gene expression, as well as phenylpropanoid and flavonoid biosynthesis, during exposure to low temperatures.

## 1. Introduction

Chilling is widely recognized as an abiotic stress that tropical and subtropical plants, including mangroves, typically encounter. Mangroves are a unique marine ecosystem possessing four key characteristics [1]. *Avicennia marina* and *Kandelia obovata*, two important mangrove species, differ in morphological traits, climatic adaptation, and geo-genetic architecture. In contrast to *A. marina*, *K. obovata* is capable of enduring a specific amount of chilling stress and is usually cultivated in regions of higher latitude. These two species are well suited for studying the response of mangrove plants to low ambient temperatures because of their different chilling sensitivities.

Following intensive studies, it has been found that exposure to low-temperature stress results in noticeable changes in the physical characteristics of plants, examples of which include leaf browning, wilting, and desiccation, as well as plant death [2,3]. Chilling activates the accumulation of reactive oxygen species (ROS), and antioxidant enzymes eliminate ROS in plants, such as superoxide dismutase (SOD), peroxidase (POD), and catalase (CAT) [4]. Moreover, mangrove plants that are sensitive to chilling exhibit noticeable alterations [5]. Previously, much of the research on chilling stress has focused on plant growth, physiological responses, survival rates, and biochemical indicators [6,7]. More recently, transcript profiling studies have investigated how plants react to chilling stress. Modifications in membrane fluidity due to cold stress affect Ca^2+^ influx and activate *COLD1/RGA1* and *OsCIPK7* [8,9]. The c-repeat binding factor (*CBF*) transcriptional pathway in *Arabidopsis* is induced by chilling stress [10]. The *AmCBF2* gene is also crucial for signal transduction in *A. marina* mangrove plants after exposure to chilling [11]. Certain investigations discovered a cold-induced upregulation of hormone-related genes, consistent with an increase in abscisic acid and salicylic acid [12], and that the *WRKY* transcription factors could contribute to the response of *Camellia oleifera* flower buds to low-temperature stress by regulating crucial genes involved in sugar metabolism [12]. According to the expressed sequence tags technology, 143 cold-related genes have been found in mangrove plants, which are involved in transcription, metabolism, energy, and photosynthesis in *K. obovata* [13]. Additionally, a recent transcriptomics analysis of various tissues in *K. obovata* found that cold-responsive differentially expressed genes originate from metabolic pathways of plant hormones, secondary metabolites, and calcium signaling [14].

Metabolites are believed to be the real drivers in the ultimate stress responses of plants, especially secondary metabolites. For example, a recent study has shown that anthocyanin accumulation altered the petal coloration of *Scutellaria baicalensis* [15]. Several free amino acids and secondary metabolites, including flavonoids and phenylpropanoids, have been identified as regulators of plant homeostasis under environmental stress conditions [16,17]. Diterpenoids have been induced in maize (*Zea mays*) and rice (*Oryza sativa*) under abiotic stress, including UV, cold, and drought stress [18,19,20]. *Cyclocarya paliurus* leaves displayed substantial flavonoid accumulation due to reprogramming of transcription factors in flavonoid biosynthesis under salt stress [21]. The phenylalanine-induced phenylpropanoid pathway was found to alleviate chilling injuries in *Mangifera indica* L. fruit during storage at suboptimal temperatures [22]. Cold-induced changes in sugar accumulation and metabolism were identified in the flower buds of *Camellia oleifera* through subsequent metabolomics investigations, which revealed the presence of a honey-like mucilaginous substance [12]. Furthermore, a recent study conducted a comparison of the chilling response between two subspecies of Asian rice (*Oryza sativa*), elucidating a rice adaptation mechanism that is dominated by reactive oxygen species [23]. Nevertheless, there is limited understanding of the metabolic-level chilling tolerance of mangrove plants, particularly concerning integrated transcriptomics and metabolomics.

Integrated transcriptomics and metabolomics technology has emerged as a prevalent research trend for investigating plant response to chilling stress. The reprogramming of gene expression and metabolic adjustment mechanisms play a crucial role in enhancing plant survival under stressful environmental conditions. In this study, we performed a comparative analysis of transcriptomics and metabolomics between *K. obovata* and *A. marina* to identify the metabolic models responsible for the chilling response. To the best of our knowledge, this combination of multiple omics is the first to be applied to chilling research in mangrove plants, and a number of genes associated with this trait have been identified. The results are expected to deepen our understanding of how to improve the resilience of mangrove plants to low temperatures.

## 2. Results

### 2.1. Phenotypic and Physiological Responses under Chilling Stress

**Figure 1:**
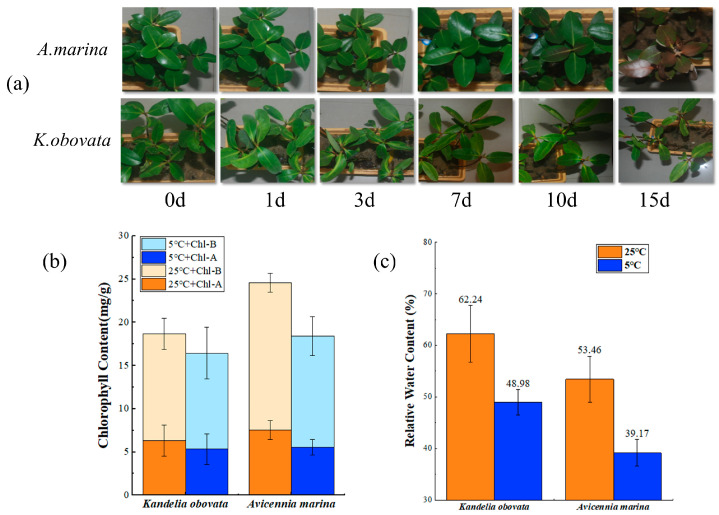
Effect of 5 °C and 25 °C treatments on phenotypic and physiological characteristics of *K. obovata* and *A. marina*. (**a**) Phenotype characteristics. (**b**) The content of chlorophyll-a and chlorophyll-b. (**c**) The content of relative water. Bars indicate the mean ± standard deviation (*n* = 3 seedlings).

After 7 days (d) of chilling treatment, the plants showed no noticeable differences in phenotype between *K. obovata* and *A. marina*. However, *A. marina* displayed an increasing browning phenotype between 10 d and 15 d of the chilling treatment, while *K. obovata* only showed mild damage throughout the 15 d chilling treatment (Figure 1a). Physiological responses demonstrated a reduction in the levels of chlorophyll-a, chlorophyll-b (Figure 1b), and water content (Figure 1c) in *K. obovata* and *A. marina* under chilling treatment. The decrease in *A. marina* was more noticeable, with a reduction of 26.44%, 24.53%, and 26.73%.

**Figure 2:**
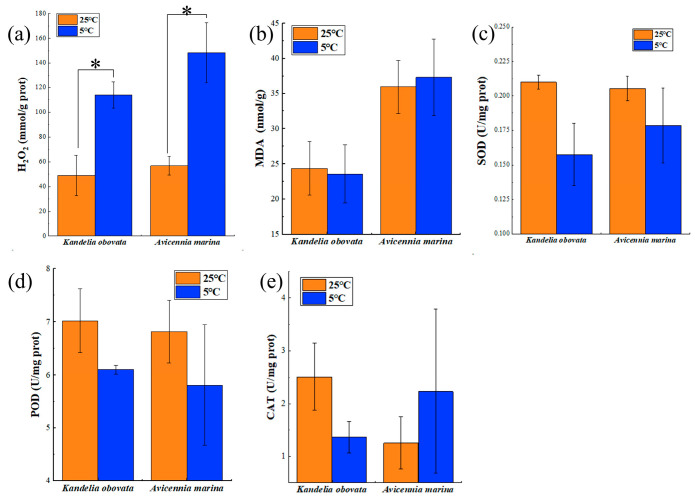
Effect of 5 °C and 25 °C treatments on phenotypic and physiological characteristics of *K. obovata* and *A. marina*. (**a**) hydrogen peroxide (H_2_O_2_) content; (**b**) malondialdehyde (MDA) content; (**c**) superoxide dismutase (SOD) activity; (**d**) peroxidase (POD) activity; (**e**) catalase (CAT) activity. Bars indicate the mean ± standard deviation (*n* = 3 seedlings). Differences were evaluated using the independent samples *t*-test (* *p* < 0.05).

Additionally, the study revealed that both *K. obovata* and *A. marina* displayed increased accumulation of hydrogen peroxide (H_2_O_2_) under chilling conditions, increasing by 1.33-fold and 1.61-fold, respectively (Figure 2a). Malondialdehyde (MDA) also increased (Figure 2b), with *A. marina* increasing by 3.87% and *K. obovata* exhibiting a decrease of 3.25%. After chilling treatments, SOD showed significantly reduced levels compared to the control group, decreasing by 13.11% in *A. marina* and 24.97% in *K. obovata* (Figure 2c). Likewise, POD exhibited lower levels compared to the control, with a reduction of 14.78% in *A. marina* and 13.12% in *K. obovata* (Figure 2d). Notably, *A. marina* exhibited higher CAT activity than the control group, with an increase of 77.83% (Figure 2e).

### 2.2. Global Transcriptional Characteristics under Chilling Stress

**Figure 3:**
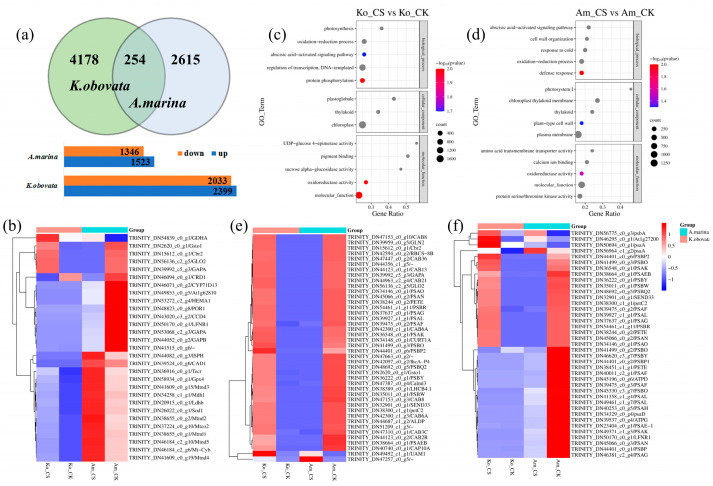
The differentially expressed genes (DEGs) and gene ontology (GO) and DEGs expression analysis. (**a**) The number of DEGs identified in two mangrove varieties in response to chilling stress. (**b**) The most significantly expressed genes with more than eightfold change in *K. obovata* and *A. marina* under chilling stress. (**c**) GO enrichment of DEGs in *K. obovata*. (**d**) GO enrichment of DEGs in *A. marina*. (**e**) The 43 DEGs co-regulated *K. obovata* and *A. marina* in the oxidation-reduction process (GO: 0055114). (**f**) DEGs co-expressed in the *A. marina* and *K. obovata* in the photosynthesis pathway.

After removing low-quality reads, we obtained 142.78 Gb of clean data for further analysis (Supporting Information Table S2). The fold changes of chosen genes exhibited distinctions between RNA-seq and qRT-PCR, although the pattern of changes remained consistent (Figure S1). A total of 8993 and 5941 DEGs were detected in *K. obovata* and *A. marina*, respectively. Significantly, partial (gene fragments) and functional (annotated functional genes) DEGs were noted, which were enriched in KEGG pathways. In *K. obovata*, we identified 4432 DEGs matching known genes. Of these, 2399 were upregulated, and 2033 were downregulated (Supporting Information Table S3). In *A. marina*, we detected 2869 matched known DEGs, with 1523 upregulated in chilling-treated groups (Supporting Information Table S3). In the KO-CK vs. KO-CS and AM-CK vs. AM-CS comparisons, upregulated DEGs were higher than downregulated in *K. obovata* than in *A. marina*. In Table S3, a Venn diagram shows that there were 254 DEGs shared by *K. obovata* and *A. marina* and 4178 and 2615 DEGs uniquely expressed in *K. obovata* and *A. marina*, respectively (Figure 3a). Among 254 shared genes, 42 genes had a response of more than eightfold to chilling stress in *K. obovata* and *A. marina*, suggesting that these genes may have a role in chilling tolerance (Figure 3b). The cluster analysis of the heat map generated two groups, with the first group containing only two DEGs, namely K12184 (TRINITY_DN47257_c0_g5) and *UAM1* (UDP-arabinopyranose mutase 1). Most of the genes shared were divided into a second group. These genes encode enzymes related to the photosynthetic process, such as chlorophyll a-b binding protein (*CAB*, *CAB6A*, *CAB8*, and *CAP10A*) and photosystem I reaction center subunit (*PSAG*, *PSAL*, and *PSAK*). In addition, under chilling stress, *K. obovata* and *A. marina* exhibited completely opposed trends in the second group pattern.

GO analysis showed that DEGs enriched in biological processes demonstrated a general response between *K. obovata* and *A. marina* under chilling stress. Among the processes were the oxidation-reduction process (GO: 0055114) and the abscisic acid-activated signaling pathway (GO: 0009738) (Figure 3c,d). In the oxidation-reduction process (GO: 0055114), 451 DEGs were involved in *K. obovata*, 306 in *A. marina*, and 43 DEGs were co-regulated in both varieties (Figure 3e). The expression of several genes, including gluconate 5-dehydrogenase (Ga5DH), glutathione s-transferase U8-like (Gsto1), NADH dehydrogenase subunit 1/2 (Mtnd1/2), and glutamate dehydrogenase 2 (GDHA), was found to be higher in *K. obovata* than in *A. marina* under chilling conditions. In contrast, copper/zinc superoxide dismutase (Sod1) and cinnamyl alcohol dehydrogenase 1 (CAD1) showed reduced expression. Certain genes displayed differential expression in *K. obovata*, namely, L-ascorbate peroxidase2/3 (APX2/3), Fe-superoxide dismutase (SODB), glutathione peroxidase 8 (GPX8), and polyamine oxidase 1 (PAO1). We tested the KEGG pathways according to *p* ≤ 0.05 of *K. obovata* (Figure S2a) and *A. marina* (Figure S2b). In *K. obovata*, phenylpropanoid biosynthesis, cysteine and methionine metabolism, and nitrogen metabolism were significantly enriched (Supporting Information Table S4). However, indole alkaloid biosynthesis and betalain biosynthesis were predominant in *A. marina*. Photosynthesis-antenna proteins and photosynthesis were enriched in *K. obovata*. In the KEGG analysis, the unique DEGs in the chilling-tolerant species *K. obovata* were classified in ABC transporters, plant hormone signal transduction, *MAPK* signaling pathway–plant and amino sugar and nucleotide sugar metabolism pathways. Overall, the results demonstrated the existence of both general and specific molecular responses to chilling stress in the two mangrove species.

### 2.3. Photosynthesis Characteristics under Chilling Stress

Photosynthesis-antenna proteins and photosynthesis were highly enriched in both *K. obovata* and *A. marina*. Upon analyzing morphological characters and chlorophyll content, *A. marina* exhibited a chlorophyll deficiency. To further examine the effects of chilling on *A. marina*, we observed the transcript abundance of key genes involved in photosynthesis-related pathways. A total of 38 DEGs associated with KEGG photosynthesis pathways were induced. This included 11 genes involved in photosystem I (PSI), 15 involved in photosystem II (PSII), 5 related to cytochrome b6/f complex, 6 related to F-type ATPase, and only 1 related to photosynthetic electron transport (Figure S3a). A total of 10 DEGs were related to KEGG photosynthesis pathways, of which 4 were involved in light-harvesting chlorophyll protein complex I (LHCI) and 6 in LHCII (Figure S3b). In *A. marina*, there was an upregulation of the *PsaA* and *PsaB* genes, while 9 *Psa*-related genes were repressed. Furthermore, the genes related to LHCI and LHCII showed decreased expression levels in *A. marina*. Additionally, 39 differently co-expressed genes were identified in *A. marina* compared to *K. obovata* (Figure 2f, Supporting Information Figure S4a,b and Table S5). Among 4 *Psa*-type genes that were upregulated in *K. obovata*, 3 identical genes were downregulated in *A. marina*. In *A. marina*, 21 DEGs were repressed in the photosynthesis pathway, while only *Psb27* was downregulated in *K. obovata*. The gene expression pattern in the photosynthesis-related pathways of *A. marina* exhibited a downregulation of the majority of DEGs, which was consistent with the reduction in chlorophyll content.

### 2.4. Key Genes in Signal Transduction Pathways under Chilling Stress

**Figure 4:**
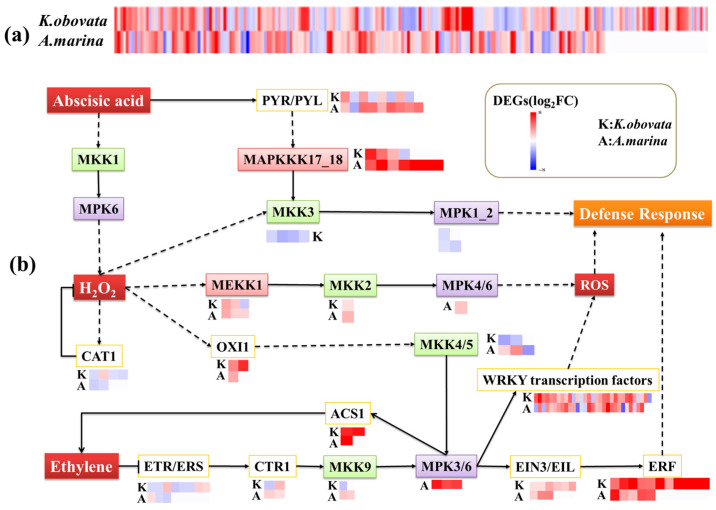
Differentially expressed genes (DEGs) for MAPK signaling pathway–plant (ko04016) in *K. obovata* and *A. marina*. DEGs encoding these genes are represented by boxes next to the gene names, and their differential expression after chilling treatment is color-coded. FC represents the fold change after chilling stress compared to the control. (**a**) All DEGs enriched in MAPK signaling pathway–plant (ko04016). (**b**) A possible signal transduction model of the chilling tolerance mechanism. After signal reception, stress-activated ROS signaling and hormone signaling modulate the MAPK signaling pathway, such as transcription factors and kinases. *PYR/PYL*: abscisic acid receptor PYR/PYL family; *MKK*: mitogen-activated protein kinase kinase; *MAPKKK17_18*: mitogen-activated protein kinase kinase kinase 17/18; *MPK*: mitogen-activated protein kinase; *MEKK1*: mitogen-activated protein kinase kinase kinase 1; *CAT1*: catalase; *OXI1*: serine/threonine-protein kinase OXI1; *ACS1*: 1-aminocyclopropane-1-carboxylate synthase 1; *ETR/ERS*: ethylene receptor; *CTR1*: serine/threonine-protein kinase CTR1; *EIN3/EIL*: ethylene-insensitive protein 3; *ERF*: ethylene-responsive transcription factor 1.

The KEGG analysis revealed that 171 DEGs were involved in the MAPK signaling pathway–plant (ko04016) in *A. marina*, while 207 DEGs were associated with chilling stress in *K. obovata* (Supporting Information Table S6). Among them, 52 and 32 DEGs were transcription factors in *K. obovata* and *A. marina*, respectively, mostly comprising *WRKY* family genes. Additionally, the *MAPK* signaling pathway also involved genes from the *MYC* and *ERF* family. Interestingly, only 9 DEGs were found to be common between these two species. Specifically, there were significant differences in gene expression between the two varieties that were analyzed. Out of the identified DEGs, 5 genes, namely calmodulin-2 (*CALM-2*), abscisic acid receptor PYL4 (*PYL4*), calmodulin-1 (*MYL12B*), nucleoside diphosphate kinase-like (*Nme2*), and calmodulin-1 (*CALM-1*), showed higher expression levels in *A. marina* compared to *K. obovata*. Significant downregulation of the *CAT1* and *CAT2*, which regulate H_2_O_2_, was found in *A. marina*. Four genes, namely calmodulin-1/2 (*CALM-1/2*), calmodulin (*Calml3*), and calmodulin-1 (*CAM-1*), which relate to calmodulin protein, were found to have participated in the MAPK signaling pathway among 9 downregulated DEGs. In addition, mitogen-activated protein kinase kinase kinase 1 (*MEKK1*), mitogen-activated protein kinase kinase 4/5 (*MKK4/5*), and mitogen-activated protein kinase 3/6 (*MPK3/6*) were also involved (Figure 4, Supporting Information Table S6). The integration of the abscisic acid and ethylene pathways involved the ethylene response factor (*ERF*) and abscisic acid receptor (*PYL*). Two varieties showed mostly upregulated DEGs encoding *ERF*. Additionally, two *MPK3* DEGs encoding *MPK3* were identified in *A. marina*. The expression level of 1-aminocyclopropane-1-carboxylate synthase (*ACS1*) involved in ethylene synthesis was upregulated in *A. marina* and *K. obovata*. DEGs encoding *WRKY* family genes were mainly upregulated in both varieties, with 26 out of 31 in *K. obovata* and 22 out of 29 in *A. marina*. Concerning mitogen-activated protein kinase, the number of identified DEGs involved was higher in *A. marina* than in *K. obovata*, including 19 genes involved in *K. obovata* and 31 in *A. marina*. Furthermore, almost all DEGs encoding mitogen-activated protein kinase were upregulated in the susceptible *A. marina* variety.

### 2.5. Metabolic Characteristics under Chilling Stress

**Figure 5:**
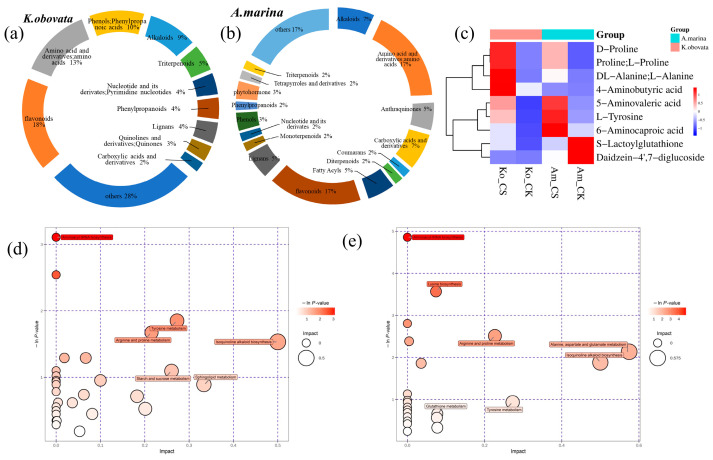
The differentially accumulated metabolites (DAMs) maps of cold-responsive metabolites. (**a**) A total of 102 DAMs were identified in chilling-tolerant *K. obovata* leaves and divided into 11 classes. (**b**) A total of 58 DAMs were obtained in *A. marina* divided into 17 classes. (**c**) A total of 9 DAMs co-induced in *K. obovata* and *A. marina*. (**d**) KEGG enrichment of DAMs in *K. obovata* and (**e**) KEGG enrichment of DAMs in *A. marina*.

A total of 102 differentially abundant metabolites (DAMs) were detected in *K. obovata* leaves tolerant to chilling stress. Flavonoids were identified as the most abundant DAMs, comprising 18% of the total metabolites (Supporting Information Table S7, Figure 5a). Of the 102 metabolites, 49 were upregulated, and 53 were downregulated. DAMs were identified. Most of the upregulated metabolites identified were amino acid derivatives such as 4-aminobutyric acid, l-tyrosine, proline, 5-aminovaleric acid, and isoleucine, as well as flavonoids including isovitexin, glycinol, and 4′,7-Isoflavandiol, and d-maltose. In the case of *A. marina*, 58 DAMs were obtained, including 20 that were upregulated and 38 that were downregulated. Among the 58 metabolites analyzed, flavonoids were found to be the most abundant (8 metabolites), followed by amino acids and derivatives (4) and alkaloids (4) (Supporting Information Table S7, Figure 5b). Moreover, it was found that *A. marina* exhibited downregulated flavonoids and lignans. A higher number of DAMs were present in *K. obovata* compared to *A. marina*, indicating that the chilling-tolerant varieties regulated more metabolites in response to chilling, which is consistent with the results of DEGs in the transcriptome. The Venn analysis demonstrated that 9 DAMs (Figure 5c) were co-induced in both *K. obovata* and *A. marina*, 93 were specific to *K. obovata*, and 49 were specific to *A. marina*. Certain metabolites, such as 4-aminobutyric acid, d-proline, proline, l-proline, and al-alanine, l-alanine exhibited a higher amount in *K. obovata* in comparison to *A. marina* under all circumstances. In contrast, daidze-in-4′,7-diglucoside and s-lactoylglutathione had a decreased quantity in *A. marina*. The widely acknowledged stress-responsive 4-aminobutyric acid (GABA) was identified as specific to chilling-responsive metabolites in two varieties, obtaining a variable importance in projection (VIP) of 2.25.

In *K. obovata*, DAMs were enriched in 31 KEGG terms (Supporting Information Table S8, Figure 5d). The terms included aminoacyl-tRNA biosynthesis, starch and sucrose metabolism, and isoquinoline alkaloid biosynthesis. On the other hand, *A. marina* had 24 KGEE terms annotated (Supporting Information Table S8, Figure 5e). In *K. obovata*, the starch and sucrose metabolism pathway was enriched, whereas it was not found in *A. marina*. The beta-fructofuranosidase (INV) and beta-glucosidase (BGlu) are crucial enzymes in the process of udp-glucose to d-glucose. Additionally, hexokinase (HXK) may assist in the phosphorylation of d-glucose. Our study demonstrated that the expression levels of *BGlu* and *INV* DEGs were higher in *K. obovata* compared to *A. marina* following exposure to chilling stress. Additionally, we observed enrichment in certain lower-level metabolisms, including biosynthesis of phenylalanine, tyrosine, and tryptophan. We conducted network-based enrichment analysis based on DAMs, revealing 9 pathways for both *K. obovata* and *A. marina* (Supporting Information Table S9, Figure S5a,b). Chilling stress impacted five amino acid pathways (alanine, aspartate, and glutamate metabolism, glycine, serine, and threonine metabolism, lysine degradation, arginine and proline metabolism, and tryptophan metabolism) out of nine in *K. obovata*. Additionally, plant hormone signal transduction was a different metabolic pathway in *A. marina*. In regard to the chilling-tolerant *K. obovata*, exposure to cold conditions resulted in the accumulation of flavonoids and phenolics, particularly flavone, hesperidin, glycinol, acetovanillone, and salidroside. There were only minimal phenotypic alterations observed.

### 2.6. Integrative Analysis of Transcriptome and Metabolome

**Figure 6:**
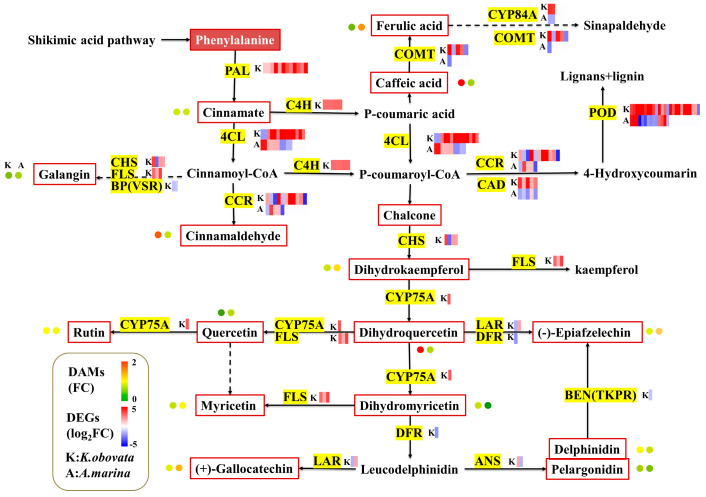
Parallel display of differentially expressed genes (DEGs) and metabolites for phenylpropanoid metabolism and flavonoid metabolism in *K. obovata* and *A. marina*. The circular pattern is the change of metabolites, from left to right, *K. obovata* and *A. marina*. Red and green colors represent increased and decreased substances, respectively. Substances in the red line were detected. The enzymes that catalyze these reactions are indicated in red, and the DEGs encoding these enzymes are represented by boxes next to the enzyme names, and their differential expression after chilling exposure is color-coded. FC represents the fold change after chilling stress compared to the control. (*POD*: Peroxidase; *CCR*: Cinnamoyl-CoA reductase; *CAD*: Cinnamyl-alcohol dehydrohenase; *PAL*: Phenylalanine ammonia-lyase; *4CL*: 4-Coumarate: CoA ligase; *FLS*: Flavonol synthase; *DFR*: Dihydroflavonol 4-reductase; *LAR*: Leucoanthocyanidin reductase; *C4H*: Trans-cinnamate 4′-monooxygenase; *CYP84A*: Ferulate-5-hydroxylate; *CHS*: Chalcone synthase; *COMT*: Caffeic acid 3-O-methyltransferase; *CYP75A*: flavonoid 3′,5′-hydroxylase; *BP*(*VSR*): vacuolar-sorting receptor 3; *BEN*(*TKPR*): anthocyanidin reductase; *ANS*: anthocyanidin synthase).

In order to investigate the genetic regulation responsible for metabolites accounting for chilling tolerance, the DEGs and DAMs were co-enriched in various KEGG pathways. Specifically, there were a total of 31 KEGG pathways that overlapped in *K. obovata* and 24 KEGG pathways that coincided in *A. marina* (Supporting Information Table S10). Moreover, it is observed that phenylpropanoid biosynthesis was ranked among the most significantly enriched pathways in *K. obovata* and *A. marina* when responding to chilling stress (Supporting Information Table S11, Figure 6). Numerous phenylpropanoid-related functional genes were discovered throughout this process, including phenylalanine ammonia-lyase (*PAL*), 4-coumarate--CoA ligase (*4CL*), cinnamoyl-CoA reductase (*CCR*), caffeoylshikimate esterase (*CSE*), caffeic acid 3-O-methyltransferase (*COMT*), and caffeoyl-CoAO methyltransferase (*CCoAOMT*). *PAL* facilitated the conversion of phenylalanine into cinnamate. Among the DEGs, 6 upregulated DEGs encoding *PAL* were identified in *K. obovata*, while no DEGs were found in *A. marina*. Similar to *PAL*, only *K. obovata* expressed a high level of *CSE*. Subsequently, cinnamate is converted to p-coumaroyl coa with the aid of cinnamic acid4 hydroxylase (C4H) and 4CL enzymes. Furthermore, the gene expression of *4CL* was more activated in *K. obovata* than in *A. marina*, apart from *PAL*s. In *K. obovata*, the expression levels of most DEGs involved in phenylpropanoid biosynthesis also increased. Additionally, 7 DEGs encoding *COMT* were induced in *K. obovata*, and 4 out of 7 were upregulated. Only one *COMT* was differentially expressed and repressed in *A. marina* exposed to chilling stress. A comparable gene expression pattern was observed for genes encoding the core enzyme of cinnamoyl-CoA reductase. Nonetheless, certain key genes showed genotype-specific variations in their expression patterns. For instance, ferulate-5-hydroxylase (*F5H*) showed upregulation in *K. obovata* but was repressed in *A. marina*.

Flavonoids are the downstream products of the phenylpropanoid metabolic pathway. The flavonoid biosynthesis pathway was uniquely identified in the leaves of *K. obovata* (Supporting Information Table S11, Figure 6). In *K. obovata* and *A. marina*, 59 and 34 DEGs were, respectively, involved in the flavonoid biosynthesis pathway (Supporting Information Table S11). Generally, nearly 40 out of 59 DEGs involved in the flavonoid biosynthesis pathway were observed to have downregulation in the tolerant variety *K. obovata*. In our study, we found that the expression of DEGs that encode significant enzymes in the flavonoid biosynthesis pathway significantly changes following chilling exposure. These enzymes included naringenin 3-dioxygenase (F3H), chalcone synthase (CHS), anthocyanidin synthase (ANS), flavonol synthase (FLS), leucoanthocyanidin reductase (LAR), bifunctional dihydroflavonol 4-reductase/flavanone 4-reductase (DFR), and flavonoid 3′,5′-hydroxylase (CYP75A). Notably, *FLS* was generally upregulated in *K. obovata* but downregulated in *A. marina* after chilling exposure. Only one DEG encoding ANR was detected, displaying differing expression patterns in two varieties and downregulated in *K. obovata*. Significant genes induced by cold exhibited similar expression profiles in *K. obovata* and *A. marina*. Only in *K. obovata* were *CHS*, *ANS*, *LAR*, *DFR*, and *CYP75A* transcriptional levels induced under chilling conditions.

## 3. Discussion

Long-term chilling may have implications for the growth, development, and geographical distribution of mangrove forests. Therefore, it is crucial to pinpoint the main mechanisms of chilling stress tolerance in mangrove plants. This study investigated the morphological characteristics and physiological indicators of *K. obovata* and *A. marina*, two mangrove varieties exposed to 5 °C chilling treatment. The findings showed that *K. obovata* exhibited a higher number of altered genes and metabolites compared to the *A. marina* group.

### 3.1. The Inhibition Effect on Photosynthesis and Antioxidants under Chilling Treatments

Chilling stress resulted in photosynthetic damage in two varieties. Previous studies revealed that short-term chilling exposure did not notably affect leaf morphology in three mangrove species (*Kandelia obovata*, *Aegiceras corniculatum*, and *Avicennia marina*) [6]. Our results concur, as both the chlorophyll and moisture levels of Pitaya (*Hylocereus* spp.) declined following cold exposure [24]. Integrative analysis of the metabolome and transcriptome indicated that chilling stress affects the process of photosynthesis in *K. obovata* and *A. marina*. The low-temperature setting hampers the absorption and utilization of light energy and carbon dioxide [25]. Chilling-tolerant varieties retain higher pigment content and photosynthetic activity compared to sensitive ones [26]. After analyzing DEGs related to photosynthesis, it was discovered that most of the DEGs were downregulated in *A. marina*. These results suggest that photosynthesis could be inhibited. Similar outcomes were observed in *Populus tomentosa*, where chilling tolerance demonstrated a superior photosynthetic capacity when exposed to chilling [27]. The biochemical reaction of photosynthesis is, indeed, sensitive to temperature and was suppressed when the temperature decreased, affecting the coupling of PSI and PSII [28].

Studies suggest that the excitation pressure of photosystem II generates ROS, including H_2_O_2_ [29]. The H_2_O_2_ content is an important indicator of ROS accumulation, and MDA content indicates the membrane lipid peroxidation [30,31]. Levels of H_2_O_2_ and MDA notably increased in *A. marina* and *K. obovata*, similar to *Avena nuda* L. and tomato plants encountering low-temperature conditions [32,33]. Antioxidant enzymes were altered in response to cold stress, including studies on *Cucumis sativus* L. [34], *Nicotiana tabacum* [35], and *Capsicum annuum* [36]. Following the chilling treatment, the antioxidant system eliminates excessive reactive oxygen species to maintain a balanced oxidative metabolism [7,37]. One of the antioxidants studied in *Oryza sativa* L., CAT, showed potential for H_2_O_2_ detoxification during chilling exposure [38]. The study concludes that the antioxidant system of the *A. marina* cannot remove excess H_2_O_2_ caused by chilling stress within cells. *APX*, known as chilling-responsive genes, encode ascorbate peroxidase, and are essential in maintaining the cell redox metabolism [39]. In the transcript analysis, two out of three *APX3* were upregulated after chilling treatment in *K. obovata*. The overexpression of *APX* improved the plant’s tolerance to chilling [40]. Some reactive oxygen genes in plants [41], such as glutathione/thioredoxin peroxidases (*GPX*) [42], glutathione S-transferases (*GST*) [43], and glutathione (*GSH*) [44], have also been identified as participating in the oxidation-reduction process in response to chilling exposure. Here, the upregulation of DEGs encoding *GST* and *GSH* in *K. obovata* suggests a correlation with chilling tolerance from the perspective of oxidation-reduction homeostasis. However, the enzyme activity was significantly diminished under chilling treatment, possibly exceeding the physiological limit of plant cells and affecting the production of related metabolites. When combined with metabolic profiles, increased antioxidants show a correlation with decreased oxidation and lipid peroxidation [22].

### 3.2. MAPK Signaling Transduction May Respond to Chilling Stress Mediated by WRKY Transcription Factors and Ethylene Signaling

The MAPK signaling pathway–plant in *K. obovata* may be associated with increased tolerance to chilling temperatures, which aligns with the impacts of cold on *Zanthoxylum bungeanum* [45]. Multiple genes play a role in the MAPK signaling pathway, including *MEKK1*, *MKK2/4*, and *MPK3/6*. *MKK2* is essential to *Arabidopsis* under cold stress, with *MEKK1* triggering its activation and the subsequent phosphorylation of *MKK2* leading to the activation of *MPK4/6* [46,47]. Additionally, the *WRKY* family comprises numerous members, with 74 found in *Arabidopsis*, as reported by [48]. In the present study, *WRKY* transcription factors were activated downstream of *MKK2*, such as *WRKY7*, *WRKY22*, *WRKY24*, and *WRKY75*. Certain *WRKY* transcription factors respond to cold in *Triticum aestivum* L. and are expressed after 15 min at 4 °C [49]. Additionally, *MEKK1* was upregulated in *A. marina* and *K. obovata* under cold stress, suggesting that it was the fundamental immune response in mangrove plants. In *Arabidopsis thaliana*, *MEKK1* has been identified as playing a role in plant innate immune responses to salt stress and cold stress and can negatively regulate the homologous gene *MEKK2*. *MEKK2*, in turn, can activate the suppressor of *MeKK1/2*, specifically the R protein summ2-mediated immune response [50]. Moreover, *OXI1* (oxidative signal-inducible 1), which encodes a serine/threonine kinase, mediated the activation of mitogen-activated protein kinases through H_2_O_2_ [51]. In our research, we observed an upregulation of *OXI1* in *A. marina* and *K. obovata*. In addition, chilling had a significant impact on the signal transduction pathways of ethylene, as evidenced by the expression of *ETR1/2* (ethylene receptor), *CTR1* (constitutive triple response 1), *EIN2* (ethylene-insensitive 2), and *ACS* (1-aminocyclopropane-1-carboxylic acid synthases). Ethylene signaling plays a crucial role in cell death, which involves *MAPK* cascades activated by *MPK3* and *MPK6* [52]. *ETR1* and *ETR2* serve as ethylene receptors and act as the upstream of *CTR1*, which encodes a negative regulator of the ethylene response pathway [53]. *CTR1* was upregulated in *K. obovata* and involved in ethylene-mediated signal transduction and defense responses under chilling stress conditions [54]. *CTR1* transmitted the ethylene signal to reach the downstream transcription factors, such as *WRKY33* [55]. Simultaneously, *CTR1* phosphorylated the downstream signal, *EIN2*, to control signaling without ethylene [56]. Interestingly, it was shown that *CTR1* repressed *MKK9* in the absence of ethylene, leading to the activation of *MPK3* and *MPK6* [57]. These two genes regulate the biosynthesis of ethylene through the catalysis of ACS [55]. In light of our findings, it is evident that MAPK signaling pathways are typically linked with hormonally-controlled developmental processes in mangrove plants.

### 3.3. Phenylpropanoid and Flavonoid Biosynthesis Contributes to Chilling Tolerance

The secondary metabolites of the phenylpropanoid and flavonoid pathways play a pivotal role in the plant stress response [21,58,59]. For example, research has shown that stress triggers the production of species-specific diterpenoids in switchgrass (*Panicum virgatum* L.) tissues [60]. In our study, we found that the pathways of phenylpropanoid biosynthesis were significantly enriched in both *K. obovata* and *A. marina*. This was evidenced by the upregulation of *PAL*, *C4H*, *4CL*, and *COMT*. Phenylpropanoid and flavonoid biosynthesis mitigate chilling injury [22]. PAL facilitates the initial stage of phenylpropanoid metabolism and stimulates the conversion of phenylalanine into cinnamate [61,62]. In the subsequent phase, 4CL catalyzes the cinnamate or p-coumaric acid. This process serves as the crucial substrate for flavonoid or H-lignin biosynthesis, the two most important metabolites branching off from phenylpropanoid biosynthesis [63,64]. *CHS* is recognized as the initial step in the flavonoid biosynthesis pathway that produces dihydrokaempferol. Moreover, the overexpression of the *SlF3HL* gene was proven to enhance the chilling tolerance of tobacco, which correlates with increased flavonoid levels [65]. In the late biosynthetic stage, DFR and LAR promote the generation of catechin. Several key genes, including *F3H*, *CHS*, *FLS*, *DFR*, and *LAR*, were notably altered under chilling treatment. Previous studies have demonstrated that plants with deficient *CCR* had decreased lignin content [66]. Additionally, phenolics played a role in the production of phenylpropanoid metabolism and served as substrates for enzymatic browning [61,67], which could be linked to browning in *A. marina* leaves. Our study discovered that *BHLH* family transcription factors (*BHLH2*, *BHLH25*, *BHLH30*, etc.) were altered, indicating that these genes established a connection with flavonoid biosynthesis in mangrove plants [68,69,70]. Based on the referring *WRKY* transcription factors, it has been reported that a positive association exists between *WRKY* genes and flavonoids, specifically *F3H*, *FLS*, *DFR*, and *ANS* in red-fleshed apples [71]. The findings imply that transcription factors influenced flavonoid biosynthesis.

## 4. Materials and Methods

### 4.1. Plant Materials, Treatments, and Sample Collection

In this study, mangrove seedlings of *K. obovata* and *A. marina* were cultivated in plastic vessels filled with soil from the locally grown seedlings. One-year-old seedlings were selected for continuous chilling treatment at 5 ± 1 °C for 10 days at a control temperature of 25 ± 1 °C in an artificial climate chamber (14/10-h light/dark cycle, 20,000 Lux during the daytime, and 70% humidity). Soil Properties: pH: 6.79 ± 0.05; salinity: 0.63 ± 0.06 g/L; total nitrogen: 2.34 ± 0.05 g/kg; total phosphorus: 1.00 ± 0.09 g/kg. The first sampling was before plants were put into the growth chamber, grouped as CK (0 days) with 6 biological replicates labeled KO_CK1 to KO_CK6; the other samples were placed in the growth chamber for 10 days and labeled KO_CS1 to KO_CS6. Each sample was divided into two parts, half for targeted metabolism detection and the other half for transcriptomic sequencing. The procedure for *A. marina* was completely the same as for *K. obovata*. The labels were AM_CK1 to AM _CK 6 and AM_CS1 to AM_CS6.

### 4.2. Physiological Parameters and Statistical Analysis

In this experiment, chlorophyll content was obtained with the ethanol extraction method. Fresh leaves were soaked in ethanol, after which their absorbance was measured at 663 nm and 645 nm using a spectrophotometer. To determine the relative water content, the initial step involves obtaining water content. Firstly, leaf samples were washed with Milli-Q water, dried at 105 °C for 30 min, and dehydrated at 70 °C until completely dry in a thermostatic dry box. The water content was then divided by the fresh weight of the leaves. The antioxidant enzyme activities of CAT, SOD, and POD were detected, along with the levels of H_2_O_2_ and MDA, utilizing commercially available assay kits (Nanjing Jiancheng Bioengineering Institute, Nanjing, China). The photo-reduction of nitroblue tetrazolium [72] was used to assay SOD. The reaction mixture contained 0.5 mL of leaf extract, 50 mM of phosphate buffer (pH 7.8), 2 mM of riboflavin, 75 mM of nitroblue tetrazolium, and 13 mM of methionine, and reacted with 120 umol m^−2^ s^−1^ light intensity for 20 min, and the absorbance was measured at 560 nm. POD was determined using the peroxidase guaiacol method [73]. Leaf extract (50 mL) was mixed with 20 mM of guaiacol and 10 mM of H_2_O_2_. One unit of POD activity was defined as catalytic production of 0.01 absorbance unit with the amount of the enzyme at 470 nm min^−1^ g^−1^ tissue sample. The UV absorption method was utilized to measure CAT [74]. One unit of CAT activity was defined as catalytic production of 0.01 absorbance unit with the amount of the enzyme at 240 nm min^−1^ mg^−1^ protein. H_2_O_2_ was measured according to the reaction product of hydrogen peroxide and molybdic acid, and the absorbance was measured at 405 nm [75]. MDA was assayed using the thiobarbituric acid chromogenic method [76]. Samples (0.5 g) were ground in trichloroacetic acid (4.5 mL, 5%), ground, and centrifuged. The reaction mixture contained the supernatant and 2 mL of 0.67% of thiobarbituric acid, which was boiled for 20 min and centrifuged. The absorbance was measured at 450, 532, and 600 nm.

The data are presented as means ± standard deviation (SD). Statistical analysis was carried out using SPSS v. 22.0 (IBM Company, Armonk, NY, USA) and Origin 2021 (Origin Lab Company, Northampton, MA, USA). Significant differences between the 5 °C chilling treatment and the control were evaluated for each time interval and marked with an asterisk (*) denoting significance (* *p* ≤ 0.05, ** *p* ≤ 0.01).

### 4.3. RNA-Seq and Statistical Analysis

Total RNA was extracted using trizol reagent (Invitrogen, Carlsbad, CA, USA) in accordance with the manufacturer’s instructions. The total RNA quantity and purity were analyzed using a Bioanalyzer 2100 and RNA 1000 Nano LabChip Kit (Agilent, Santa Clara, CA, USA). The quality of each RNA sample was ensured. Biotree (Shanghai, China) carried out transcriptomic sequencing and database construction. The initial data were thoroughly filtered by eliminating low-quality reads and reads with adapter and poly-N to yield pure reads. The raw transcript data will be archived in the NCBI SRA database, (https://dataview.ncbi.nlm.nih.gov/object, accessed on 1 October 2024), or please contact corresponding author. The analysis relied on clean data. Each gene’s FPKM was then calculated based on its length and the number of mapped reads. The DESeq R package (1.18.0) was employed for differential expression analysis. The resulting *p*-values were adjusted using Benjamini and Hochberg’s method to regulate the false discovery rate. Genes with an adjusted *p*  <  0.05 and |log_2_ fold change| ≥ 1 were defined as DEGs. Statistical analyses were conducted in R, and plots and heatmaps were created using the ggplot2 and pheatmap packages in R statistical software (R version 4.0.5, R core team). Gene ontology (GO) databases were used to annotate gene function with default parameters (http://www.geneontology.org, accessed on 1 October 2024). KEGG pathway enrichment was carried out using MetaboAnalyst 5.0’s pathway analysis module (https://www.metaboanalyst.ca/home.xhtml, accessed on 1 October 2024), which is based on the Kyoto Encyclopedia of Genes and Genomes (KEGG, https://www.genome.jp/kegg/pathway.html, accessed on 1 October 2024).

### 4.4. qRT-PCR Verification

qPCR was carried out using the applied biosystems steponeplustm real-time system with the kit of SYBR premix wx taqtm II reagents (Takara, Kyoto, Japan). Each qRT-PCR reaction was repeated three times, and the investigation data were analyzed using the 2^−ΔΔCT^ method. The internal reference gene of *K. obovata* was *GAPDH*, and for *A. marina*, it was *Actin*. The gene-specific primers used for qRT-PCR are listed in Supporting Information Table S1, based on transcriptome findings. Data are presented as SD. Statistical significance was determined using SPSS. Asterisks (*) indicate significant differences evaluated for each time interval between the 5 °C chilling treatment and its control (* *p* ≤ 0.05, ** *p* ≤ 0.01).

### 4.5. Metabolomics Analysis

Clean leaf tissues were sampled from two varieties that underwent different conditions: chilling (CS) or non-chilling (CK). Biotree (Shanghai, China) conducted a widely targeted metabolomics analysis, extracting metabolites. The exionlc system (sciex) was used for UHPLC separation. Peak detection and annotation were performed using an inner R program and database. Principal component analysis (PCA) and partial least squares discriminant analysis (PLS-DA) were conducted with the R package ropls version 1.6.2. The VIP parameter was used to determine the relative importance of each metabolite in the PLS-DA model. Metabolites with significantly different expressions were defined using a combination of the VIP value (threshold > 1) and the *t*-test *p*-value (*p*  ≤  0.05) of the OPLS-DA model and were combined to define metabolites with significantly different expressions. Metabolic pathway analysis was carried out using the KEGG database and GO database. Subsequently, KEGG analysis was employed to elucidate the relationship between DEGs and metabolic pathways in a pathway-based analysis.

## 5. Conclusions

In this study, we explored the mechanism underlying the differential ability of *K. obovata* and *A. marina* leaves to tolerate cold temperatures during the seedling stage by investigating changes in metabolites and genes. The study compared gene expression levels in *K. obovata* and *A. marina* under chilling stress. The MAPK signaling pathway–plant was found to be enriched with unique DEGs in chilling-tolerant *K. obovata*, suggesting a potential relation between signal transduction and chilling tolerance. Based on the compared gene expression of DEGs in response to chilling treatments and phenylpropanoid metabolite changes, we identified several important DEGs involved in phenylpropanoid metabolism in cold-response processes, including *PAL*, *4CL*, *CCR*, *CSE*, *COMT*, and *CCoAOMT*. We also identified several synthase enzyme genes involved in flavonoid biosynthesis, including *F3H*, *CHS*, *ANS*, *FLS*, *LAR*, *DFR*, and *CYP75A*. Compared to the *A. marina* group, more DEGs were induced in *K. obovata*, which was consistent with the metabolite trend. These results imply that chilling stress triggers the establishment of cold-tolerance mechanisms, ultimately improving the adaptability of *K. obovata*. The findings of this study will contribute to revealing the self-protection mechanism of *K. obovata* in response to chilling stress at the seedling stage and further exploration of cold acclimation related genes for mangrove breeding.

## Data Availability

Data are contained within this article. Research manuscripts reporting RNA-seq datasets are deposited in the NCBI SRA database. If further information is required, please contact yswang@scsio.ac.cn.

## Supplementary Materials

The supporting information can be downloaded at https://www.mdpi.com/article/10.3390/ijms242316989/s1.
