# Supplementary material for: Comparative Transcriptomics and Metabolomics Analyses of Avicennia marina and Kandelia obovata under Chilling Stress during Seedling Stage
Source: Int J Mol Sci. 2023 Nov 30;24(23):16989. doi: 10.3390/ijms242316989 (PMC10707264; doi:10.3390/ijms242316989)
Supplement: Supplementary file 1 [file ijms-24-16989-s001.zip › ijms-2636215-supplementary.pdf]

The caption of supplement files:

Figure S1: The expression levels key genes in *K.obovata* and *A.marina* as determined by qRT-PCR.

Figure S2: KEGG pathways according to $p \leq 0.05$ of *K.obovata* (a) and *A.marina* (b).

Figure S3: The photosynthesis pathways (a) and photosynthesis-antenna proteins (b) from KEGG database (https://www.kegg.jp/pathway/map00195) of *A.marina*.

Figure S4: The photosynthesis pathways (a) and photosynthesis-antenna proteins (b) from KEGG database (https://www.kegg.jp/pathway/map00195) of *K.obovata*.

Figure S5: Network-based enrichment analysis was constructed according to DAMs in *K.obovata* (a) and *A.marina* (b). The factors include metabolic pathways, module, enzyme, reaction, compound and input compound (DAMs).

Table S1: Primers for qRT-PCR.

Table S2: Overview table of transcription sequencing quality.

Table S3: 4432 and 2869 DEGs matching known genes were detected in *K.obovata* and *A.marina*, respectively, based on $p < 0.05$, and the absolute $\log_2$fold change ≥1.

Table S4: 137 and 136 KEGG (Kyoto Encyclopedia of Genes and Genomes) pathways in *K.obovata* and *A.marina*, were enriched based on KEGG analysis, respectively.

Table S5: DEGs enriched in Photosynthesis pathway in *K.obovata* and *A.marina*, respectively.

Table S6: DEGs enriched in MAPK signaling pathway-plant in *K.obovata* and *A.marina*, respectively.

Table S7: A total of 102 and 58 DAMs were identified in *K.obovata* and *A.marina*, respectively.

Table S8: KEGG analysis of DAMs.

Table S9: Network-based enrichment analysis, including metabolic pathways, module, enzyme, reaction, compound and input compound (DAMs).

Table S10: The DEGs and DAMs were co-enriched in 31 KEGG pathways in *K.obovata*, and 24 KEGG pathways coincided in *A.marina*.

Table S11: The DEGs associated with phenylpropanoid biosynthesis in *K.obovata* and *A.marina*, including phenylpropanoid biosynthesis, flavonoid biosynthesis and phenylalanine metabolism.
