# Supplementary material for: Comparative Transcriptomics and Metabolomics Analyses of Avicennia marina and Kandelia obovata under Chilling Stress during Seedling Stage
Source: Int J Mol Sci. 2023 Nov 30;24(23):16989. doi: 10.3390/ijms242316989 (PMC10707264; doi:10.3390/ijms242316989)
Supplement: Supplementary file 1 [file ijms-24-16989-s001.zip › Table S1.pdf]

Table S1 Primers for qRT-PCR

Gene Name<br>基因名称	Primer Sequence(5’→3’)<br>引物序列（5’→3’)
AmActin-F	AGATGGCAGAAGCTGAGGA
AmActin-R	CCATGCCAACCATCACACC
Ko-GAPDH-F	TCGTCCAGGTCTCCAAG
Ko-GAPDH-R	AGCCAGATCCACCACTC
