# Supplementary material for: Comparative Transcriptomics and Metabolomics Analyses of Avicennia marina and Kandelia obovata under Chilling Stress during Seedling Stage
Source: Int J Mol Sci. 2023 Nov 30;24(23):16989. doi: 10.3390/ijms242316989 (PMC10707264; doi:10.3390/ijms242316989)
Supplement: Supplementary file 1 [file ijms-24-16989-s001.zip › Table S2.pdf]

Table S2 Overview table of transcription sequencing quality

Sample	Valid_Bases	Valid%	Q20%	Q30%	GC%
Am_CK_1	5.63Gb	97.93	98.14	94.28	46.66
Am_CK_2	5.56Gb	97.95	98.17	94.40	47.48
Am_CK_3	5.83Gb	98.09	98.17	94.37	46.55
Am_CK_4	5.79Gb	98.18	98.19	94.44	46.23
Am_CK_5	5.69Gb	97.24	97.97	93.86	46.31
Am_CK_6	6.36Gb	97.86	97.98	93.88	46.09
Am_CS_1	5.91Gb	96.97	97.87	93.64	47.61
Am_CS_2	6.16Gb	97.24	97.96	93.88	49.35
Am_CS_3	5.71Gb	96.77	97.88	93.73	50.63
Am_CS_4	6.46Gb	97.34	97.95	93.81	46.69
Am_CS_5	6.38Gb	97.61	97.90	93.70	47.55
Am_CS_6	5.93Gb	96.86	97.92	93.77	47.21
Ko_CK_1	5.61Gb	97.98	97.95	93.83	45.84
Ko_CK_2	5.84Gb	98.00	97.98	93.91	45.80
Ko_CK_3	5.97Gb	97.97	97.92	93.73	45.58
Ko_CK_4	6.20Gb	98.07	97.98	93.88	45.66
Ko_CK_5	6.09Gb	98.03	97.94	93.77	45.74
Ko_CK_6	6.20Gb	98.02	97.98	93.88	45.74
Ko_CS_1	6.10Gb	97.98	97.88	93.66	45.85
Ko_CS_2	5.97Gb	97.92	97.91	93.75	45.70
Ko_CS_3	5.93Gb	98.10	98.02	94.05	45.91
Ko_CS_4	5.50Gb	98.22	98.05	94.12	46.05
Ko_CS_5	6.16Gb	98.18	97.99	93.99	46.54
Ko_CS_6	5.80Gb	98.20	98.06	94.15	46.71
