# Supplementary figures and images for: Comparative Transcriptomics and Metabolomics Analyses of Avicennia marina and Kandelia obovata under Chilling Stress during Seedling Stage
Source: Int J Mol Sci. 2023 Nov 30;24(23):16989. doi: 10.3390/ijms242316989 (PMC10707264; doi:10.3390/ijms242316989)

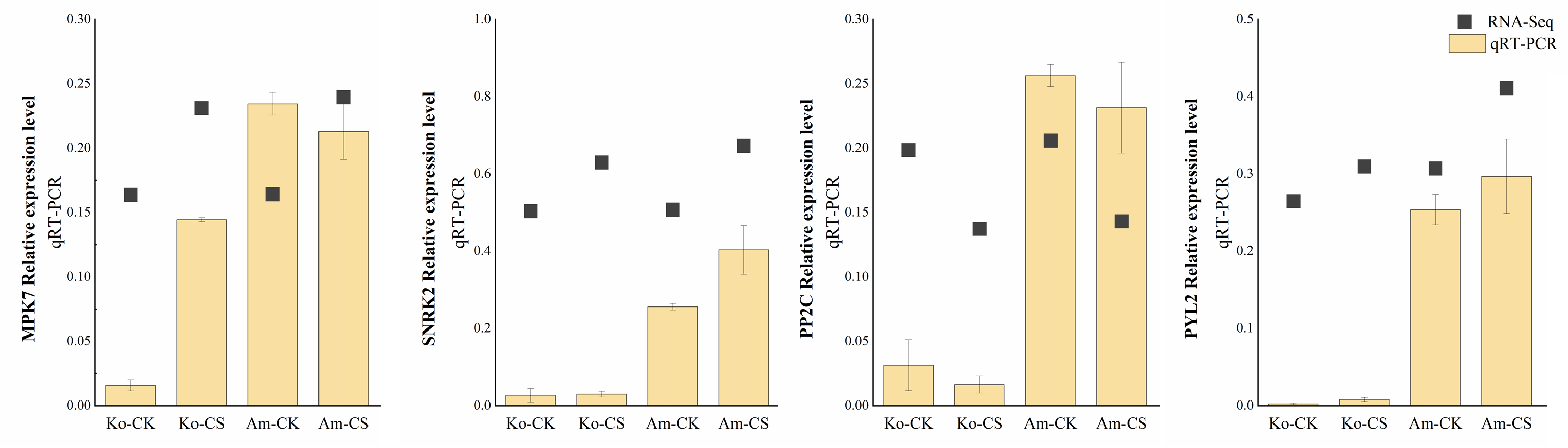

Supplement: Supplementary file 1 [file ijms-24-16989-s001.zip › Figure S1.tif]

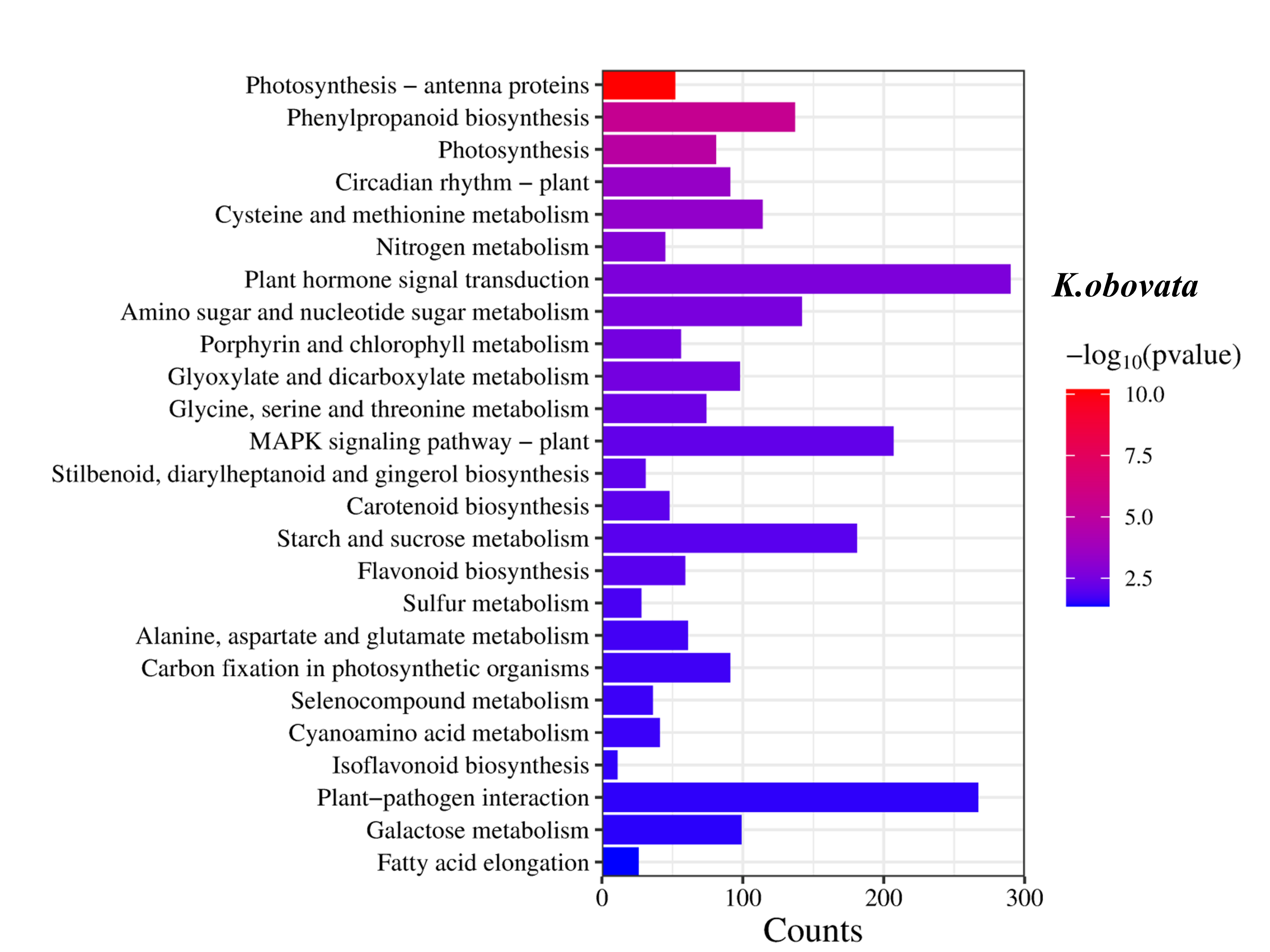

Supplement: Supplementary file 1 [file ijms-24-16989-s001.zip › Figure S2a.tif]

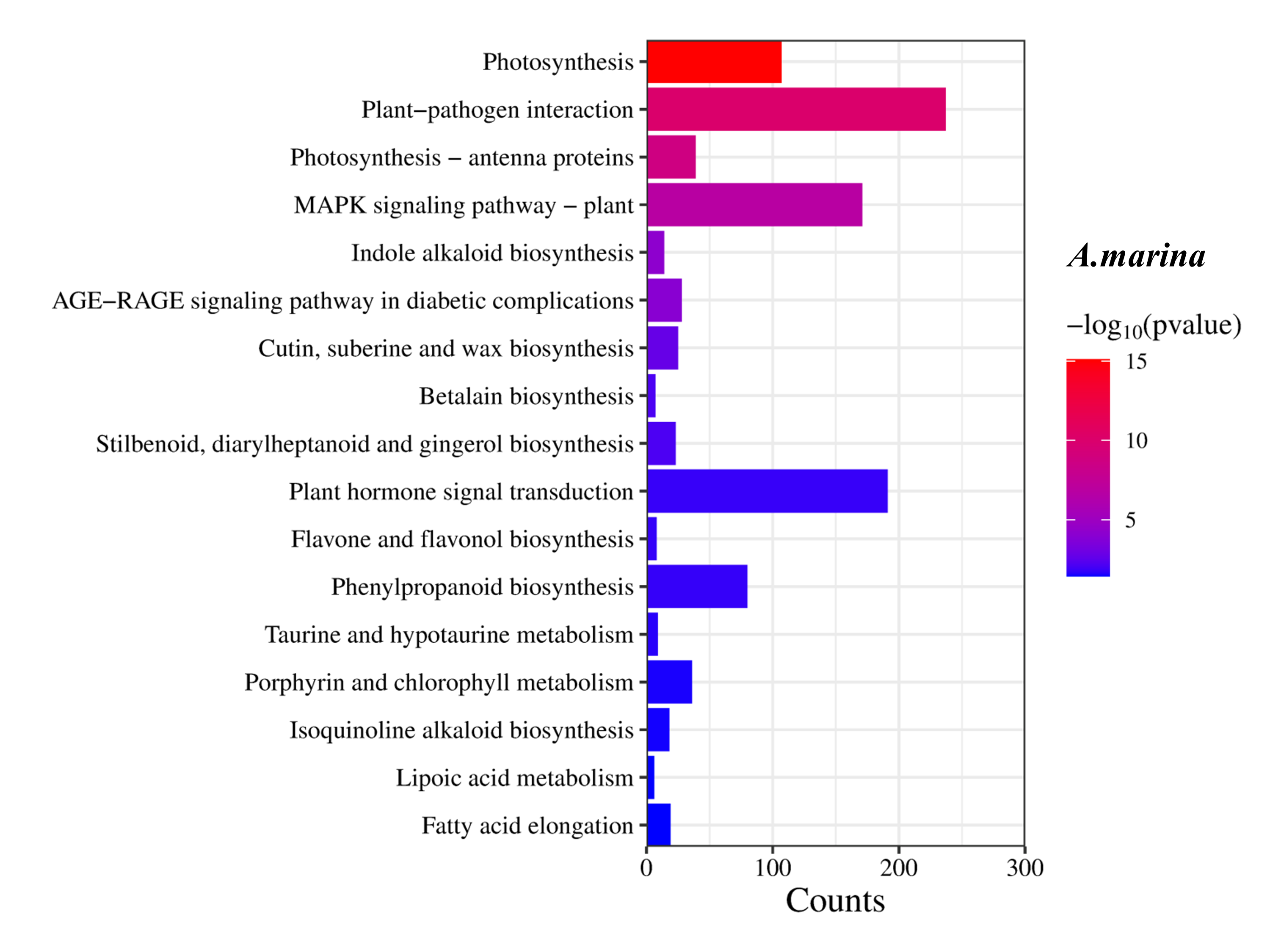

Supplement: Supplementary file 1 [file ijms-24-16989-s001.zip › Figure S2b.tif]

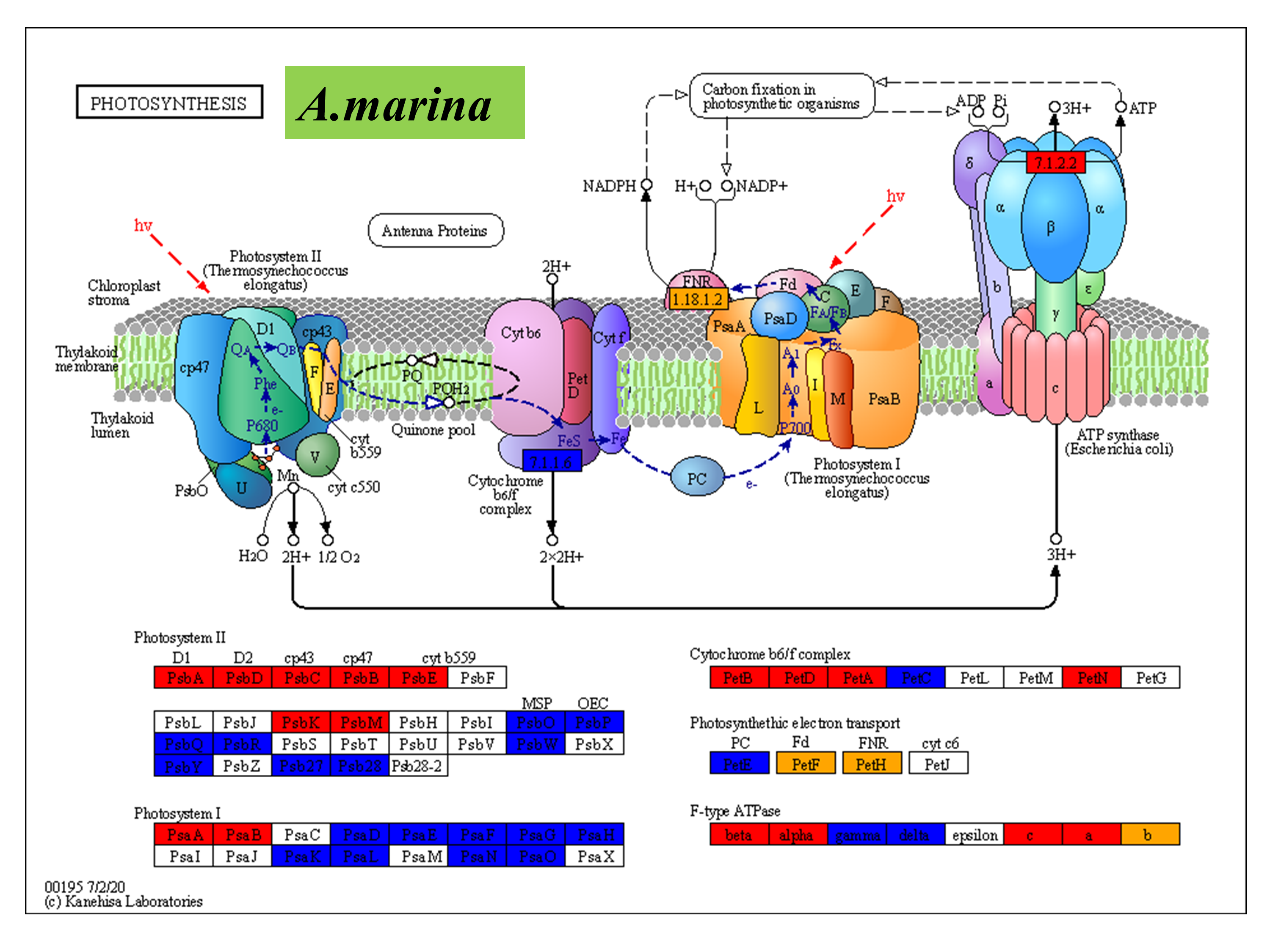

Supplement: Supplementary file 1 [file ijms-24-16989-s001.zip › Figure S3a.tif]

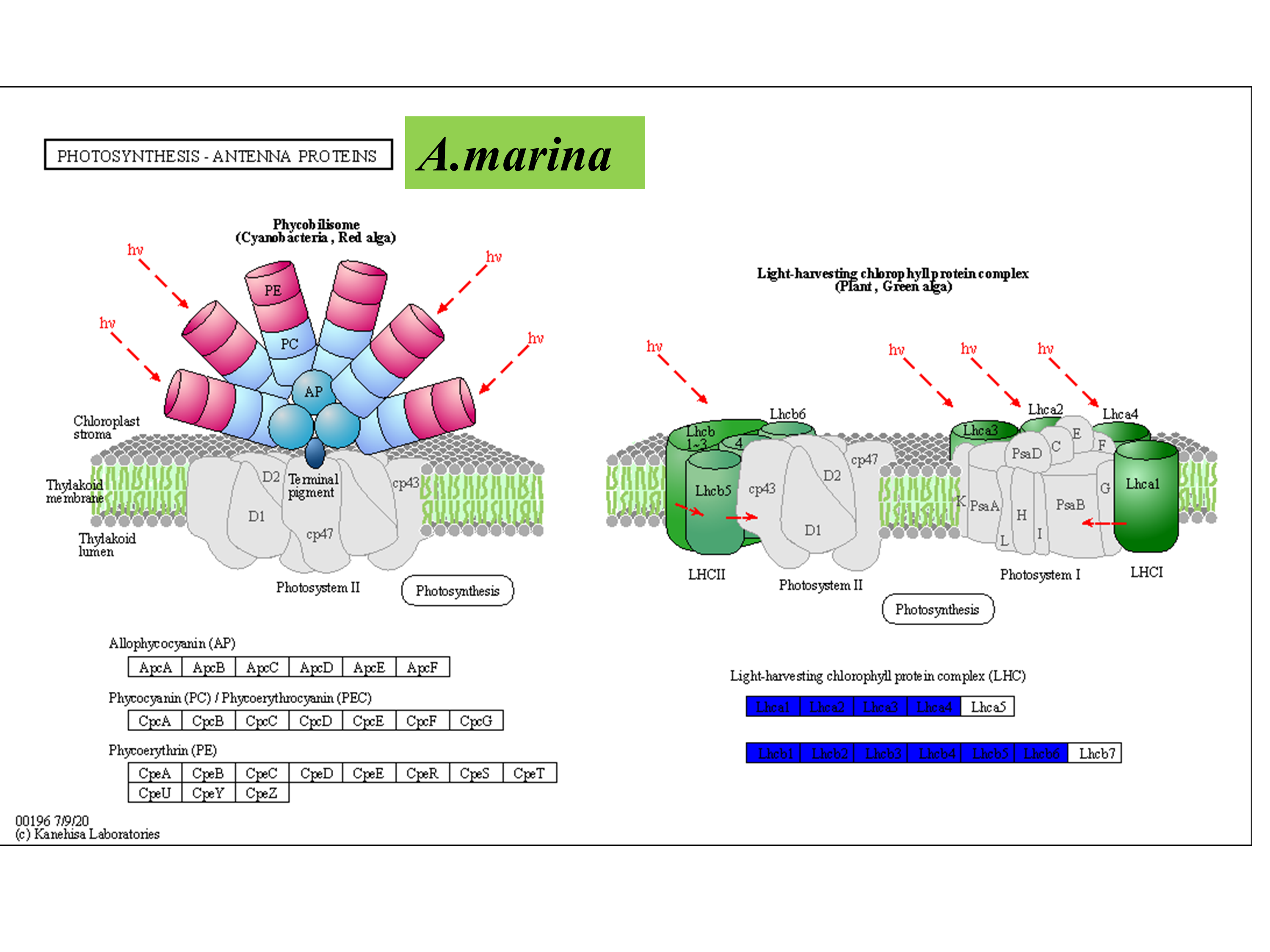

Supplement: Supplementary file 1 [file ijms-24-16989-s001.zip › Figure S3b.tif]

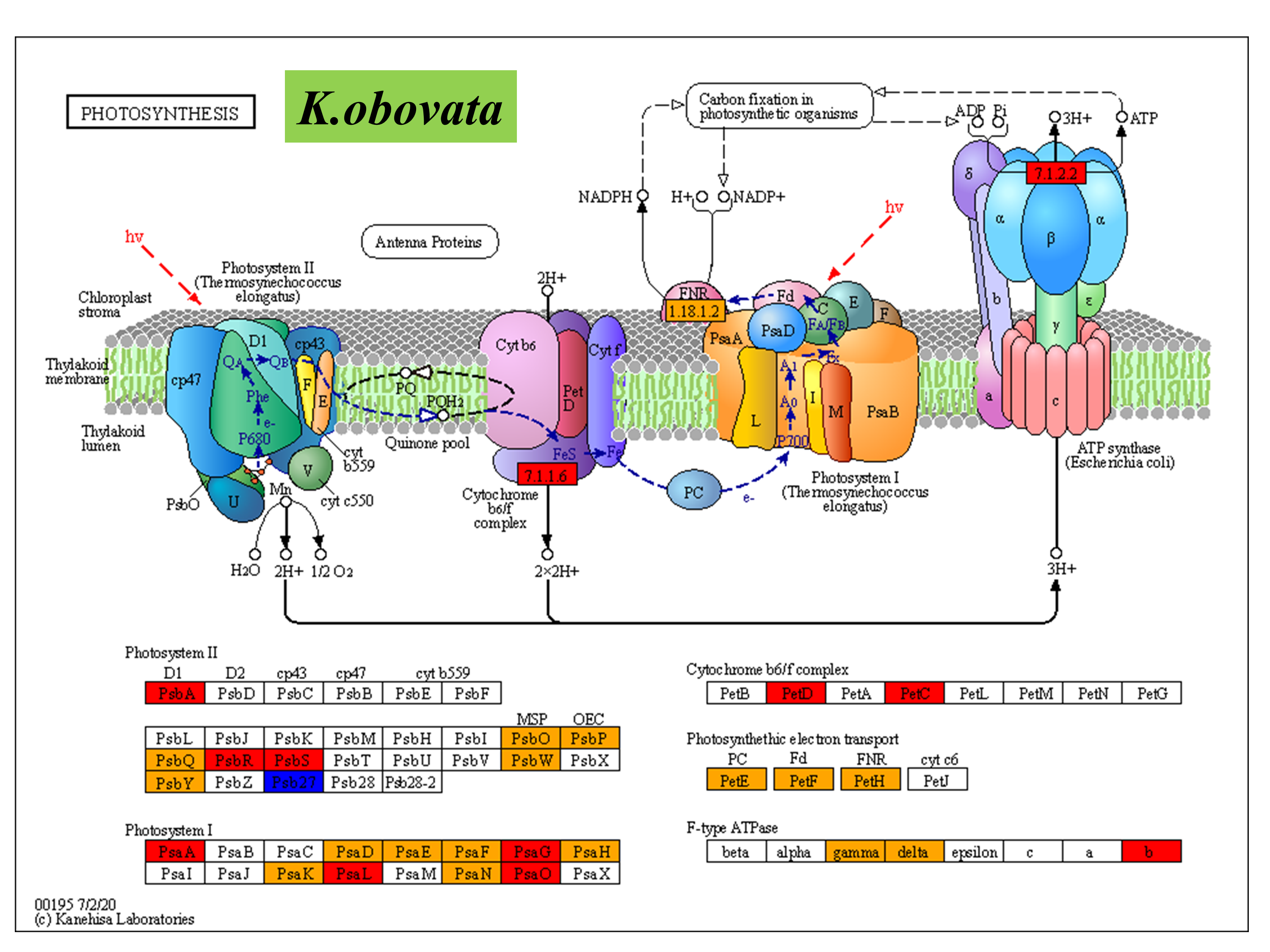

Supplement: Supplementary file 1 [file ijms-24-16989-s001.zip › Figure S4a.tif]

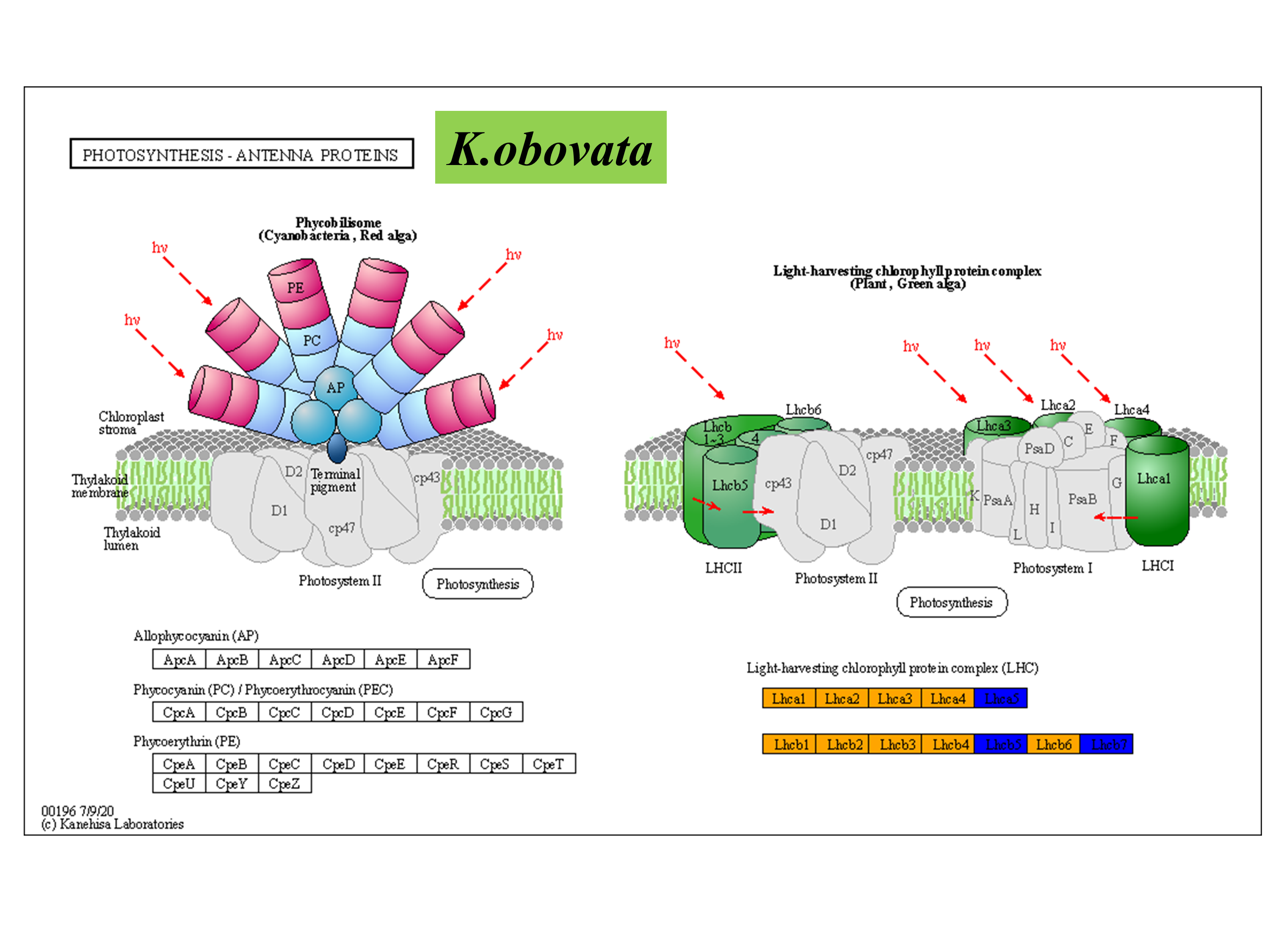

Supplement: Supplementary file 1 [file ijms-24-16989-s001.zip › Figure S4b.tif]

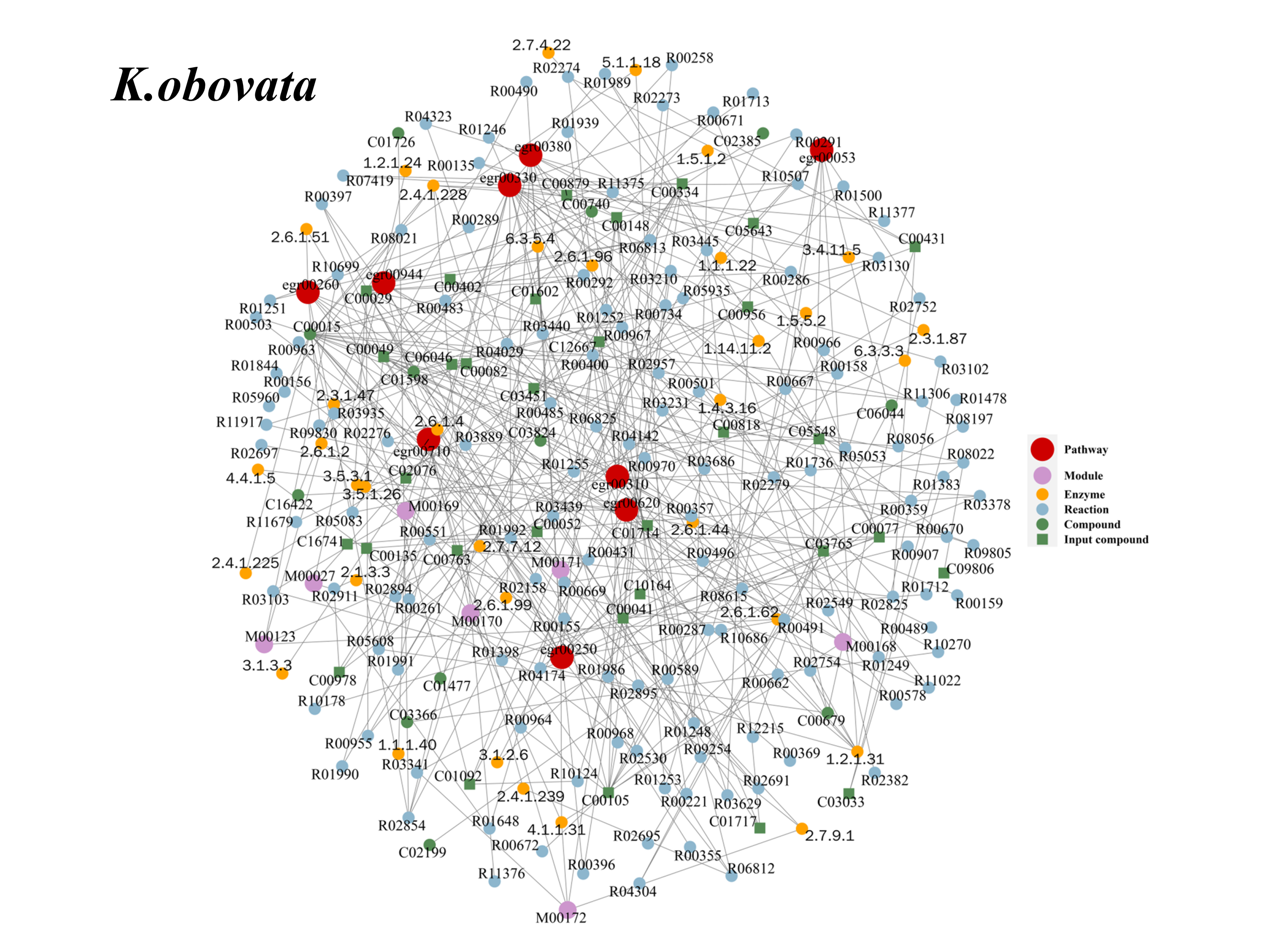

Supplement: Supplementary file 1 [file ijms-24-16989-s001.zip › Figure S5a.tif]

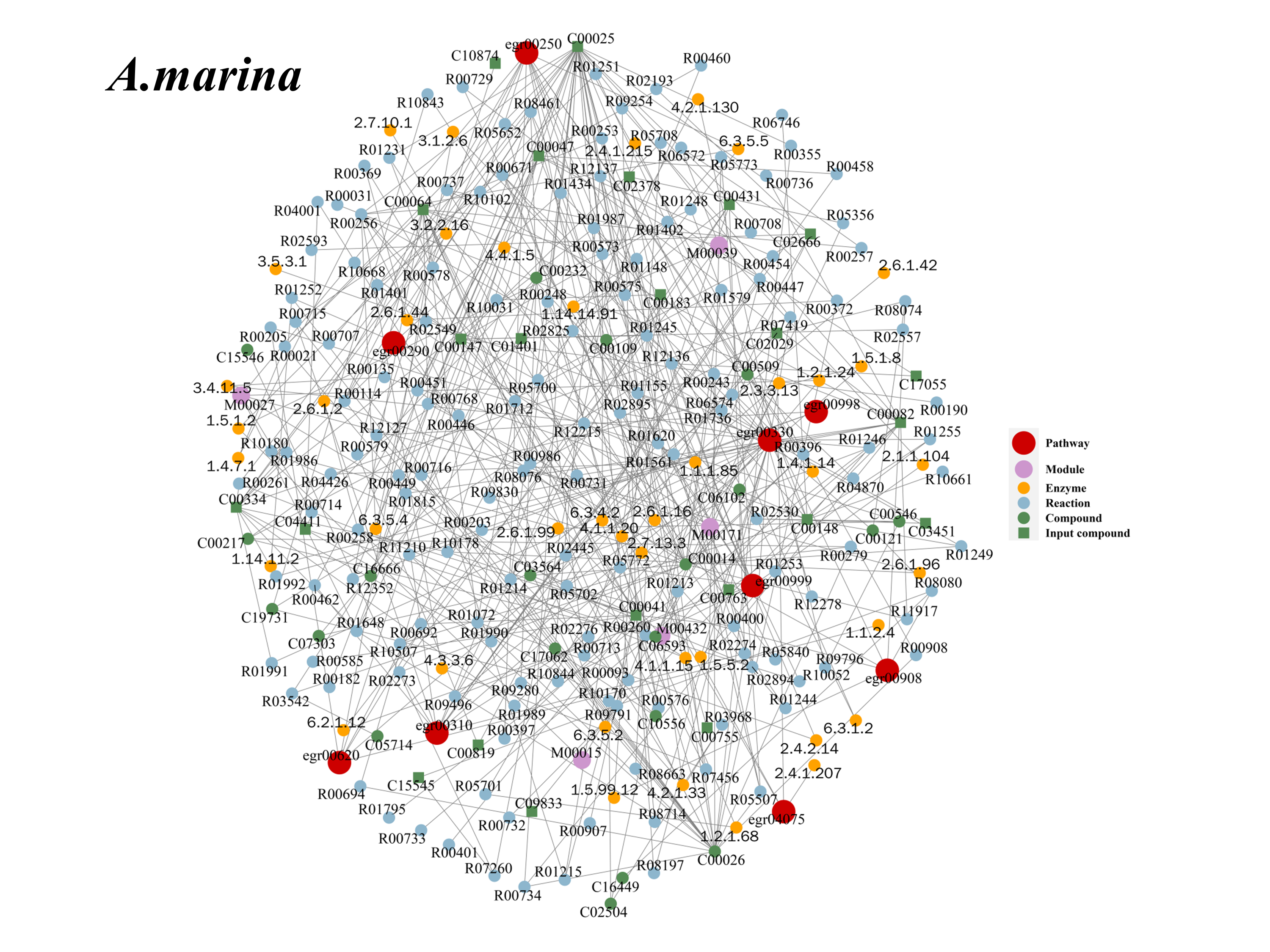

Supplement: Supplementary file 1 [file ijms-24-16989-s001.zip › Figure S5b.tif]
